# Pure Insular Cortex Infarct in Sepsis-Induced Hypoxic Ischemic Encephalopathy

**DOI:** 10.7759/cureus.20634

**Published:** 2021-12-23

**Authors:** Atif Ahmed, Eric J Basile, Myra Ahmad, Jeffrey R Blair, Hamzah Ahmad, Salman Ahmad, Patricia E Roche

**Affiliations:** 1 Physical Medicine and Rehabilitation, Nova Southeastern University Dr. Kiran C. Patel College of Osteopathic Medicine, New York, USA; 2 Internal Medicine, Touro College of Osteopathic Medicine, New York, USA; 3 Radiology, Touro College of Osteopathic Medicine, New York, USA; 4 Internal Medicine, Idaho College of Osteopathic Medicine, Meridian, USA; 5 Neurology, Touro College of Osteopathic Medicine, New York, USA; 6 Orthopaedic Surgery, Touro College of Osteopathic Medicine, New York, USA; 7 Radiology, Stony Brook University, New York, USA

**Keywords:** pediatric neuro radiology, neurological sign, brain hypoxia, brain infarct, multiple organ failure, hypoxic-ischemic encephalopathy, neonatal sepsis

## Abstract

Each year there are an estimated 1.7 million adults in the United States that develop sepsis and nearly 16% of these adult patients die because of this disease process. Sepsis, however, can impact patients of all ages. Neonatal sepsis is currently one of the leading causes of morbidity and mortality among neonates. There are many complications of neonatal sepsis including meningitis, seizures, and hypoxic ischemic encephalopathy (HIE).

HIE is estimated to impact one to five in 1000 live births worldwide, primarily impacting neonates. It is more commonly seen in premature infants and infants with low birth weights due to immature organ systems and a lack of adequate auto-regulatory mechanisms that would otherwise manage brain perfusion. In premature neonates, the most commonly recognized pathological pattern found on MRI is focal non-cystic white matter injury. HIE can also impact term infants as well. In these neonates, there exist two common MRI patterns that include either basal ganglia-thalamus ischemia, most often involving deep gray nuclei and perirolandic cortex, or watershed predominant ischemic changes that involve cortical gray matter.

We report a 38-week-old male neonate born at gestation diagnosed with HIE secondary to neonatal sepsis with an MRI finding of isolated insular cortex hypersensitivity on fluid-attenuated inversion recovery (FLAIR) and T1-weighted imaging. Isolated insular cortex hypersensitivity can be seen in non-lacunar ischemic middle cerebral artery (MCA) territory strokes but it is not common for it to present as a sole finding. In our case, these findings persisted for several weeks without evidence of any common patterns of hypoxia-induced cerebrovascular insult on MRI imaging.

## Introduction

Per the United States Centers for Disease Control and Prevention (CDC), 1.7 million adults will develop sepsis annually, with a near 20% mortality rate overall, and a whopping 33% mortality rate in the hospital setting [1]. Sepsis carries a grim economic burden, as it is the most expensive healthcare problem in the United States, needing more than $20 million in 2011 alone. Sepsis has been regarded as a medical emergency in which the body’s systemic response to an infection can result in end-organ dysfunction and ultimately death [2]. ­­Sepsis can attack at all stages of life, and neonatal sepsis is one of the leading causes of morbidity and mortality among infants. Neonatal sepsis is the body’s systemic response to an infection involving the bloodstream in newborn infants less than 28 months old [3]. If there is prompt treatment, neonatal sepsis can be resolved without any lasting issues. However, there are some possible complications including infant death, meningitis, seizures, and hypoxic-ischemic brain injury.

Hypoxia-induced cerebrovascular injuries are typically found in the germinal matrix of these preterm and low birth weight neonates. The germinal matrix is highly vascularized and may have an inherent fragility during development, leaving it prone to hemorrhage. Infarcts of these periventricular areas generally occur in the watershed zones, due to these areas having poor perfusion and less tolerability to fluctuations in cerebral blood flow [4].

There are two main patterns of brain injury in a hypoxic ischemic insult, the basal ganglia-thalamus pattern and watershed predominant pattern [5]. The watershed predominant pattern includes the vascular watershed zones, anterior middle cerebral artery (MCA) and posterior MCA, which mainly affect white matter. This type of brain injury can result in a hypoxic ischemic encephalopathy (HIE), which can be broken down into three clinical stages as per the Sarnat and Sarnat staging system: mild, moderate and severe [6]. Stage 1 (mild) is characterized by hyper-alertness, uninhibited Moro and stretch reflexes, sympathetic effects, normal EEG, and duration less than 24 hours. Stage 2 (moderate) is characterized by obtundation, hypotonia, strong distal flexion, multifocal seizures, and requires EEG should a periodic pattern be preceded by delta activity. Finally, Stage 3 (severe) is seen as stuporous, flaccid, and suppressed brain stem and autonomic functions with EEG showing infrequent periodic discharges [7]. Here we report HIE of a full-term, 38-week, neonate presenting with a pure insular cortex infarct.

## Case presentation

the patient was an infant aged 38 4/7 weeks, large for gestational age (LGA), who required body cooling and was admitted to rule out HIE. The infant was delivered by Caesarean section due to placenta abruptio, and the hospital course proceeded as such: the infant presented with severe hypoxia at birth. Initial arterial blood gas (ABG) showed lactic acidosis and severe acidemia. The patient received normal saline boluses twice in the delivery room, and dopamine and epinephrine for a week. Initial echocardiograms showed persistent pulmonary hypertension (PPHN) and a large non-restrictive patent ductus arteriosus (PDA) showing bidirectional flow, with PPHN resolving and PDA also resolving at a later date. 

The infant received antibiotics for 5 days for suspected sepsis, as blood and urine cultures were negative. Urinalysis (UA) indicated urinary tract infection (UTI) when a second treatment of antibiotics was administered, and a stress dose of hydrocortisone was also given. 

The infant was intubated in the delivery room, was placed on a conventional ventilator and extubated to nasal continuous positive airway pressure (CPAP) on DOL (day of life) 11, and weaned to nasal cannula on DOL 17, and eventually to room air on DOL 18. The patient remained on room air with saturation greater than 95%.

The infant was NPO (*nil per os*) for 10 days, with central and peripheral lines placed for blood draw and parenteral nutrition. The infant was given total parenteral nutrition (TPN) for 16 days. Feeding began on DOL 11 and stopped two days later due to hyperkalemia, which was managed by kayexalate, lasix and bumetanide. No dialysis occurred. TPN continued on DOL 14 until DOL 23, where bolus feeds began. The infant had poor nipple feeding and weight gains. Other electrolyte derangements were hyponatremia and hypercalcemia. 

Direct bilirubin was as high as 29.5 and aspartate aminotransferase (AST) levels as high as 3288, with direct bilirubin of 2.2 at discharge, managed with phenobarbital and ursodiol. Evidence of gallbladder sludge and heterogeneous liver parenchyma was noted on ultrasound (US), however, repeat US revealed normal liver size and echogenicity without sludge seen in the gallbladder. 

Hematocrit was 27.6% at birth as the infant had severe anemia, thrombocytopenia and coagulopathy-DIC (disseminated intravascular coagulation) related to HIE and side effects of hypothermia therapy, which was terminated early. The lowest hematocrit was 22%, hemoglobin was 7 g, platelets were 15,000, and fibrinogen was measured at 69 mg. The highest international normalized ratio (INR) recorded was 4.5. The infant received 11 transfusions of packed red blood cells (pRBCs), 30 of platelets, 19 of fresh frozen plasma (FFP) and three of cryoprecipitate over the first 10 days of life. Vitamin K was also given. At discharge, hematocrit improved to 34.8%, reticulocytes to 1.5%. Total/direct bilirubin at the peak was 37/26.1 which also improved to 3.5/2.2 at discharge. Infant hematology also reported A-positive, Coomb’s negative. 

The infant also had non-oliguric acute kidney injury (AKI) with the highest creatinine level of 2.67. The patient was treated with fluid resuscitation, bumetanide and electrolyte replenishment PRN (*pro re nata*). Neurology was consulted for HIE as there was a risk of seizure, however, no clinical seizure was noted, and EEG was normal. 

Brain US revealed left grade I intraventricular hemorrhage (IVH) and right choroid plexus cyst, with MRI showing insular hyperintensities on fluid-attenuated inversion recovery (FLAIR) and T1 consistent with HIE. The patient was discharged to follow-up with nephrology, gastrointestinal (GI), endocrinology, cardiology, ophthalmology, neurology and high-risk clinic, along with ursodiol, bicitra and PolyViSol medications.

**Figure 1:**
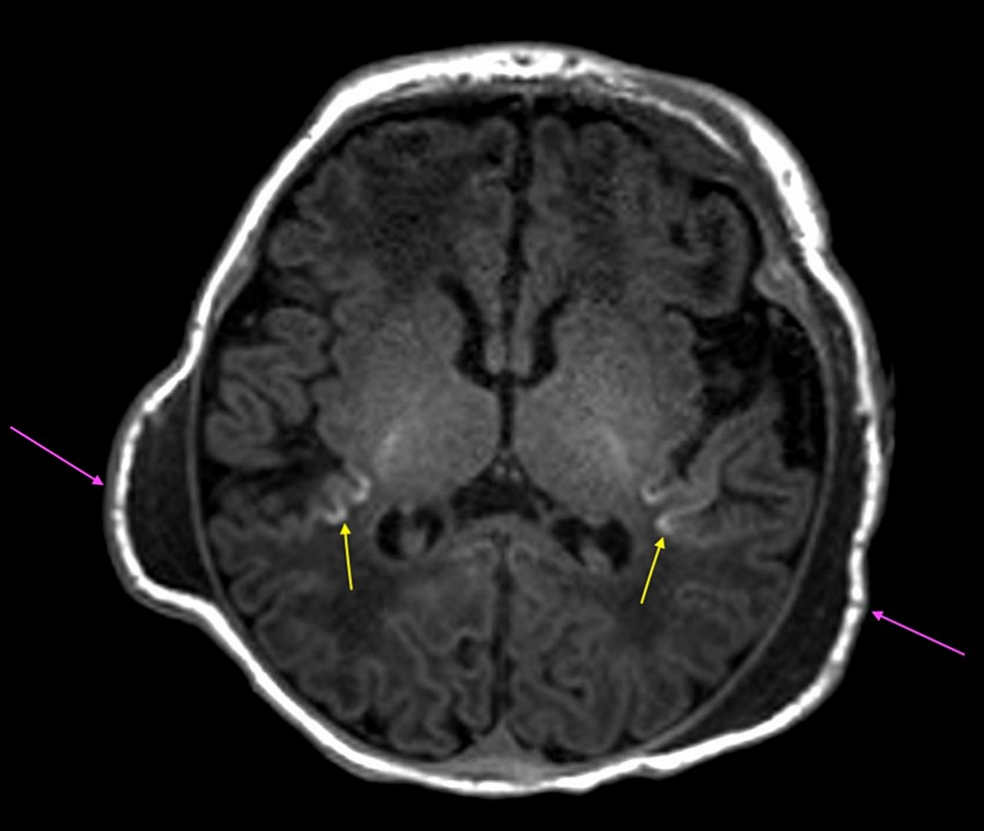
Axial T1 demonstrating signal intensity at insular cortices bilaterally (yellow arrows) along with bilateral parietal cephalohematomas (purple arrows).

**Figure 2:**
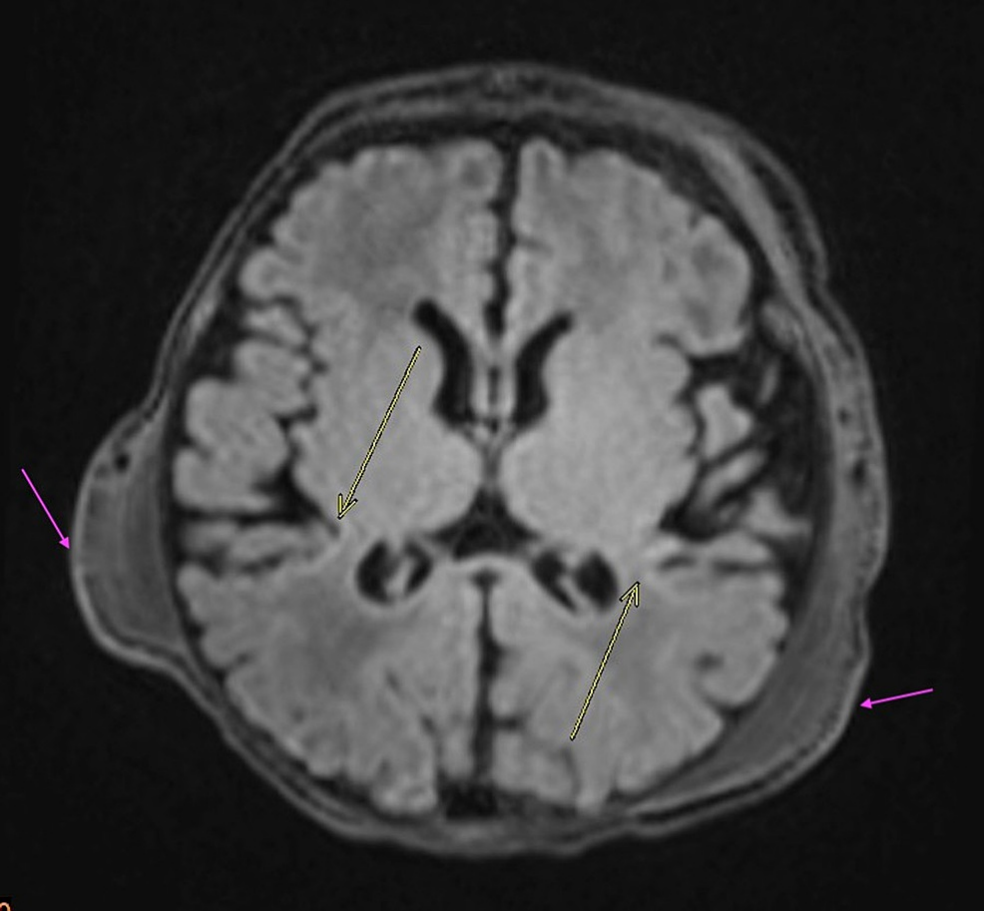
Axial T2 FLAIR demonstrating subtle increased intensity involving posterior insular cortices bilaterally (yellow arrows) along with bilateral parietal cephalohematomas (purple arrows). FLAIR: Fluid-attenuated inversion recovery

MRI of the brain without contrast revealed subtle increased signal intensity on FLAIR sequences involving the posterior insular cortex bilaterally (Figures 1, 2). T1-weighted sequences showed increased signal intensity at this location as well, revealing cortical highlighting (Figure 1). These findings were found to be consistent with the setting of neonatal hypoxic ischemic encephalopathy.

**Figure 3:**
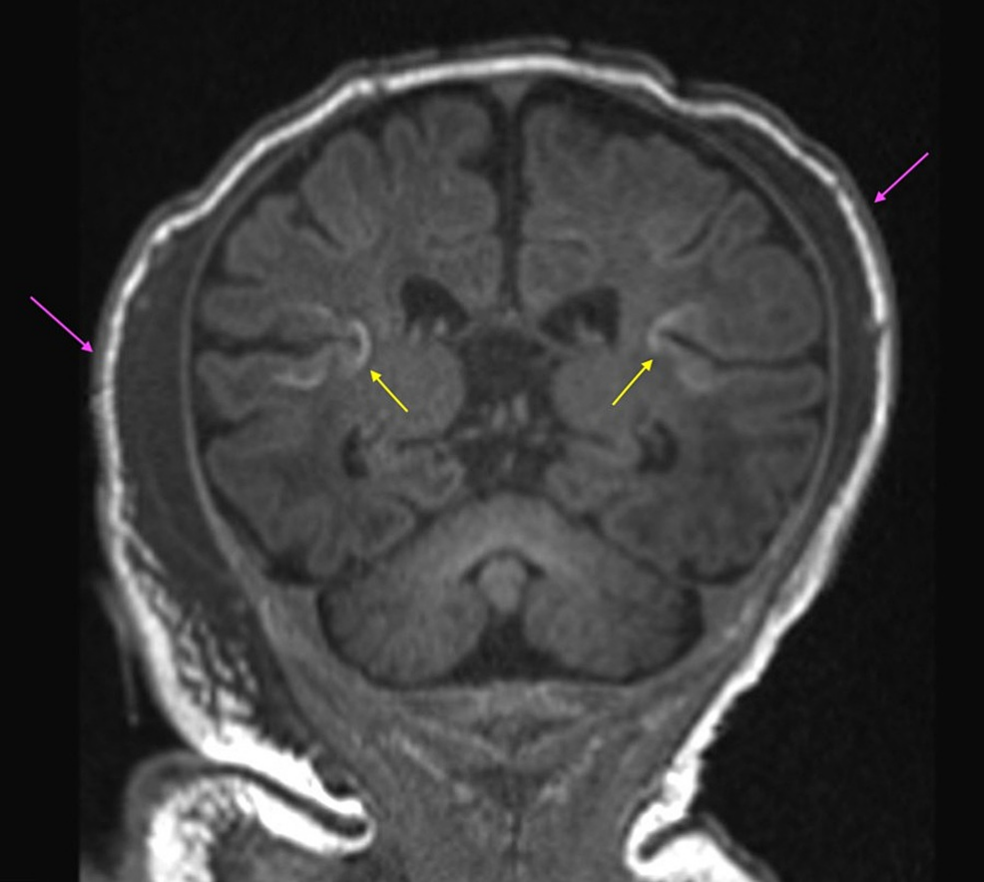
T1 coronal sequence demonstrating cortical highlighting at the insular cortices bilaterally (yellow arrows), suggestive of neonatal HIE. Bilateral parietal cephalohematomas were also appreciated (purple arrows). HIE: hypoxic ischemic encephalopathy

**Figure 4:**
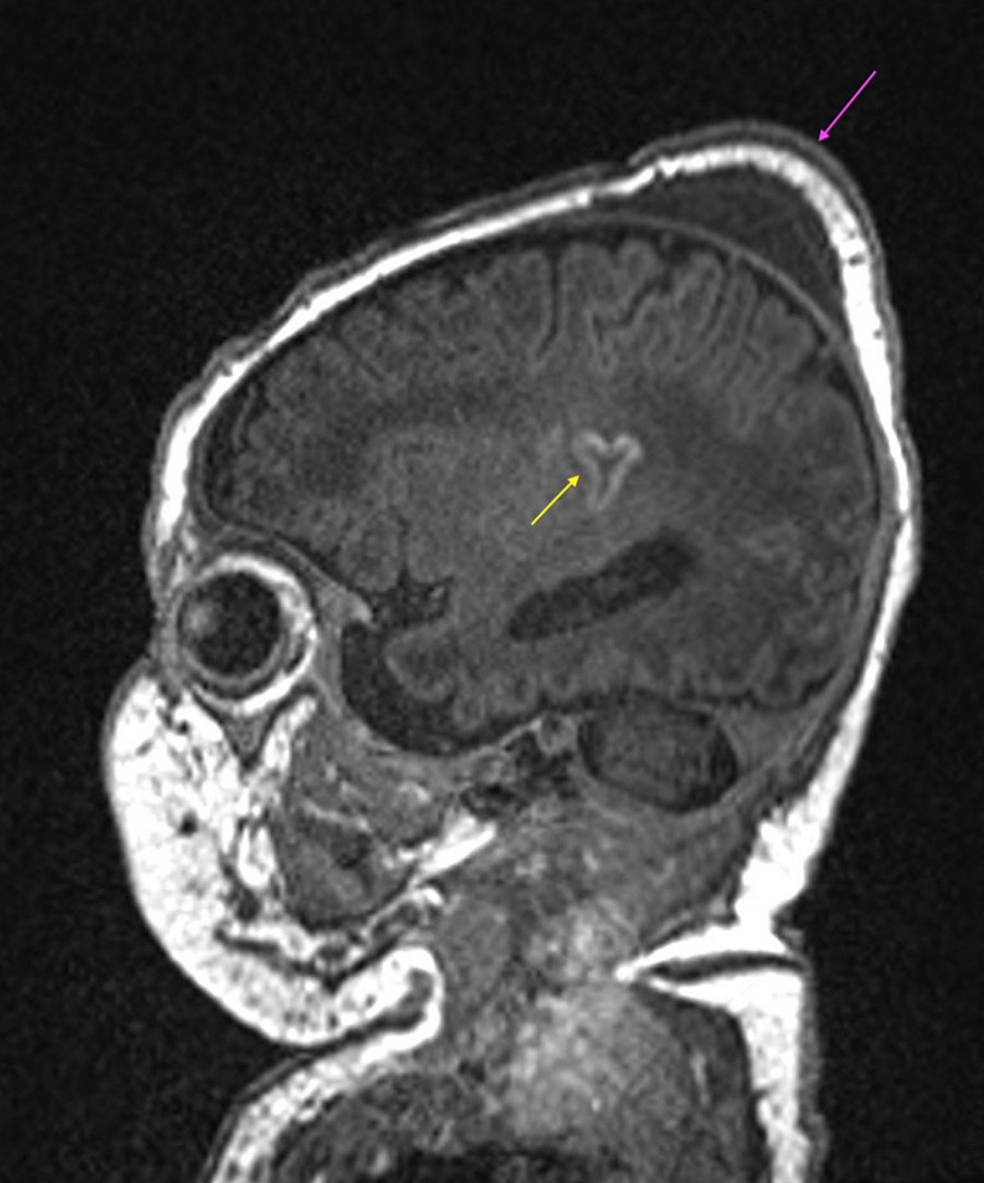
Sagittal T1 demonstrating cortical highlighting at the insular cortex (yellow arrow). Apical cephalohematoma also noted (purple arrow).

In addition, bilateral parietal cephalohematomas were present, along with an apical cephalohematoma (Figures 1-4), although no depressed skull fracture or intracranial hemorrhage was noted.

## Discussion

HIE is a significant contributor to neonatal disease burden, often with prolonged and severe complications; the condition is estimated to affect anywhere from 1 to 5 in 1000 live births worldwide and has a high correlation with the development of cerebral palsy, epilepsy, and cognitive disabilities [8]. It is primarily a condition that affects neonates -- though there is a disproportionate proclivity to affect premature infants as well as those with low birth weights [9]. The proposed reason for this is attributed to immature organ systems and autoregulatory measures to provide adequate perfusion and oxygen off-loading to cerebral tissue. Etiologies include hemorrhage -- often originating in the highly vascularized germinal matrix, true hypovolemia, hypoxia, anemia, respiratory distress, and neonatal sepsis as was the case in our patient.

This ischemic damage results in various radiographic patterns of injury, which vary by age, severity, imaging technique, and timing. There are generally three major types of lesions implicated in HIE: periventricular leukomalacia, basal ganglia thalamic lesions, and multicystic encephalopathy. In term babies with HIE, there is a bimodal distribution of ischemic damage patterns that manifests as either basal ganglia-thalamus predominant ischemic changes or watershed predominant ischemic changes. Basal ganglia predominant patterns often involve the deep gray nuclei and perirolandic cortex, while the watershed pattern often extends to involve the cortical gray matter [4]. In the premature population, focal non-cystic white matter injury is recognized as the most common pattern of hypoxic brain injury [10].

MRI has been deemed the standard neuroimaging technique to assess for perinatal asphyxia-associated brain injury in full-term infants. Further, although cranial ultrasound (CUS) has been a reliable diagnostic modality for the detection of cystic periventricular leukomalacia in preterm infants, MRI is superior to CUS in detecting diffuse and more subtle forms of white matter injury [11]. Diffusion-weighted imaging (DWI) is sensitive for the detection of injury in the first 24 hours, during which time conventional T1- and T2-weighted imaging may appear normal; thus if MRI is negative in the first 24 hours, a repeat examination should be performed again at days 2-4 to help rule out the possibility of delayed injury. It is important to note, however, that DWI performed at this time will often underestimate the full extent of injury [12]. By the second day, hyperintensities on both T1- and T2-weighted images begin to appear. As time progresses, DWI becomes less reliable as pseudonormalization of brain parenchyma occurs, which can falsely indicate an apparent resolution of signal intensity abnormalities. Thus, conventional T1 and T2-weighted MR images from day 1 are less useful than DWI but become increasingly sensitive as the insult has time to manifest. In general, T1- and T2-weighted MRI is most diagnostically appropriate at the end of the first week after the abnormalities on DWI have pseudonormalized.

Radiographic patterns of ischemic change leading to HIE are not congruent between the full-term and premature demographics. In full-term babies, deep gray matter structures are actively myelinating, which renders them areas of high energy demand and thus more susceptible to ischemia. The locations also contain the highest concentrations of N-methyl-D-aspartate (NMDA) receptors at term which predisposes them to excitotoxicity. After hypoxic insult, DWI typically displays signal hyperintensity in the ventrolateral thalami, basal ganglia, perirolandic regions, and corticospinal tracts within the first day. The same regions begin to demonstrate T1- and T2-weighted hyperintensity and more subtle changes often become noticeable by day two; the hippocampi and dorsal brainstem are also often implicated on T1- and T2-weighted MRI by the second day. T2-weighted imaging typically shows shortening in the thalami and posterior putamina by the second week of insult. T1 shortening in the thalami, basal ganglia, and perirolandic cortex can be seen for up to a few months’ duration. Early T1 shortening, if preceding T2 shortening, is often thought to be a sign of hemorrhage [12].

In the preterm demographic, injury to the thalami, basal ganglia, hippocampi, cerebellum, and corticospinal tracts are common -- the most frequently involved regions being the thalami, anterior vermis, and dorsal brainstem [6]. However, involvement of the basal ganglia in preterm neonates is not as pronounced as is the case in full-term infants. This is attributed to the fact that the basal ganglia tend to cavitate and shrink without scarring in neonates born before 32 weeks gestation. Additionally, the basal ganglia’s resistance to ischemic damage in the preterm setting can also be attributed to the relatively late myelination compared to that of the thalami; as the thalamus and globus pallidus myelinate around 25 weeks’ gestation, while the putamen and caudate myelinate at 35 weeks, susceptibility to ischemic damage in this population lies within the former. This may also explain the perirolandic cortex’s relative resistance to ischemic damage as it myelinates at 35 weeks [12].

The emerging therapies under investigation include moderate whole-body/head hypothermia, erythropoietin, hematopoietic stem cell transplant from umbilical cord blood, cannabinoid agonists, xenon, docosahexaenoic acid (DHA), and antiepileptic medications such as topiramate and phenobarbital [13]. So far, only whole-body/head hypothermia has displayed mortality and morbidity benefit in infants with Stages 2 or 3 HIE. This modality requires a target temperature goal of 33.5˚C for the first 6 hours after birth to 72 hours [14]. Hypothermia is believed to reduce free radicals, glutamate levels, oxygen demand, and apoptosis -- all contributors to ischemic and reperfusion damage responsible for HIE [13]. Symptomatic management with acidosis correction, respiratory support, fever control, and seizure prophylaxis also fall under the focus of treatment.

In our case, the male neonate was born at 38 weeks gestation to a mother who underwent emergent Cesarean section for placenta abruptio. The infant had prolonged poor Apgar scores -- 1, 3, 4, 6, and 6 at 1, 5, 10, and 15 minutes, respectively. Serum studies demonstrated a low cord pH, initial hemoglobin of 7.8, and an elevated lactic acid concentration. Two blood cultures had no growth for 48 hours, urine culture was negative, and methicillin-resistant *Staphylococcus aureus *(MRSA) culture was negative. A diagnosis of hypoxic ischemic encephalopathy secondary to neonatal sepsis was made, and the neonate was transferred to the neonatal ICU (NICU) after resuscitation. He was placed on head cooling, treated for sepsis, and then experienced disseminated intravascular coagulopathy complicated by subsequent multiorgan failure. Initial MRI findings displayed isolated insular hyperintensities on FLAIR and T1-weighted imaging. The remaining brain parenchyma was unremarkable, and no common patterns of hypoxia-induced cerebrovascular insult were appreciated. Serial T1-weighted imaging showed a persistent insular cortical highlighting on the third day of life with maximum signal occurring on the second week. The signal persisted for several weeks, and no other abnormalities were appreciated. The neonate ultimately succumbed to his illness.

The persistent, isolated insular cortex hyperintensity secondary to HIE has not been well documented in the available literature. Insular cortex involvement is commonly implicated in patients with non-lacunar ischemic MCA territory strokes in the general population, but even so, it rarely presents as the sole finding on MRI. In cases where it has been involved in ischemic strokes, it has been associated with larger infarcts and poorer prognosis [15]. As the insular cortex is responsible for autonomic autoregulatory mechanisms of homeostasis, it would not be unreasonable to suspect it as a contributor to the neonate’s septic clinical manifestation and subsequent multiorgan failure. If it is the case that the insular lesion contributed to the neonate’s multi-organ failure, it highlights even further the need for continued research as well as improved imaging modalities in the setting of HIE. It would be of tremendous benefit to the community for correlations between imaging abnormalities and clinical outcomes to be made and perhaps influence medical decisions. Moreover, due to the high incidence of morbidity associated with HIE, investigation into prevention and treatment should be prioritized.

## Conclusions

HIE is estimated to impact one to five in 1000 live births worldwide, primarily impacting neonates. It is more commonly seen in premature infants and infants with low birth weights due to immature organ systems and a lack of adequate auto-regulatory mechanisms that would otherwise manage brain perfusion. In premature neonates, the most commonly recognized pathological pattern found on MRI is focal non-cystic white matter injury. HIE can also impact term infants as well. In these neonates, there exist two common MRI patterns that include either basal ganglia-thalamus ischemia, most often involving deep gray nuclei and perirolandic cortex, or watershed predominant ischemic changes that involve cortical gray matter. Currently, whole-body/head hypothermia has been the only therapy to show any benefit in mortality and morbidity in neonates with HIE stage 2 or 3. With respect to our case, we report a 38-week-old male neonate diagnosed with HIE secondary to neonatal sepsis with an MRI finding of isolated insular cortex hypersensitivity on FLAIR and T1-weighted imaging. These findings persisted for several weeks without evidence of any common patterns of hypoxia-induced cerebrovascular insult on MRI imaging. This case demonstrates an abnormal MRI finding of a neonate diagnosed with HIE. Further investigation is therefore warranted into the correlation between uncommon MRI findings and clinical outcomes for patients with HIE. Understanding this correlation will undoubtedly lead to a greater ability to accurately and promptly diagnose HIE to then deliver whole-body/head hypothermia sooner rather than later during a neonate’s clinical course. 
